# De novo HNF1A mutation of young maturity-onset diabetes 3 of a young girl—Case report

**DOI:** 10.1186/s12902-023-01293-7

**Published:** 2023-02-14

**Authors:** Haoran Peng, Jianbo Li, Zhang Wang

**Affiliations:** 1 Chengdu Medical College, 610083 Chengdu, China; 2 Southwest Medical University, 646000 Luzhou, China; 3 Department of Geriatrics, The General Hospital of Western Theater Command, 610083 Chengdu, China

**Keywords:** MODY3 child Sulfonylurea Compounds

## Abstract

Young maturity-onset diabetes of the young type3(MODY3) as a special type of diabetes, the probability of diagnosis is low. This article reports on a case and reviews the relevant knowledge of the disease. We report an 11-year-and-11-month-old girl whose grandmother died from diabetic complications while the rest of the families were non-diabetes. The proband was initially treated with insulin and metformin but the threatment proved inefficient. After an exome-targeted capture sequencing test, she was diagnosed with mature-onset diabetes of young type 3 (MODY3), and sulfonylureas make sense. The key to mody treatment is a correct and timely diagnosis, which contributes to helping patients overcome the problems of MODY3, especially for blood sugar control.

## Background

MODY was first found in the 1970s, and 14 subtypes were identified. MODY3 is the most common subtype of MODY. The typical age of onset is 6 months to 35 years [1]. MODY3 results from the pathogenic effect of gene mutations (high allelic heterogene -HNF1A) characterized by progressive insulin secretion defect, decreased renal threshold for glucose reabsorption, lean, on-autoimmunity mediated, onset of non-Insulin-dependent diabetes mellitus (NIDDM) at an early age. This autosomal dominant onozygotic form of diabetes has significant fasting and prandial hyperglycemia, sensitivity to sulfonylurea therapy, and frequent development of end-organ complications (including microvascular and macrovascular complications) and liver adenomatosis in rare families. The clinical manifestation of HNF1A-MODY varies greatly, even within one family, such as polyuria and polydipsia.

The prevalence of Mody is 50–100/million [1], and the specific proportion of HNF1A-MODY varies by country and region, with 0,0066% in Croatia [2] and 3.6% in the UK [3].The proportion of MODY3 in Mody also changes a lot. Overlapping features constantly lead to misdiagnosis as T1DM or T2DM. Therefore, the article aims to help physicians improve their diagnostic awareness of mody3.

## Case presentation

An 11-year-and-11-month-old girl was referred to us from the endocrinology clinic on 26 August 2021, due to hyperglycemia complaints of polyuria and polydipsia for more than ten days. She was found to have hyperglycemia after an accident resulting in a fracture of the left femoral neck more than 8 months ago. At that time, her fasting plasma glucose was 8.9mmol/L, postprandial glucose was 12.0mmol/L, and glycated hemoglobin (HbA1c) was 6.7%, but without symptoms of diabetes. Without hypoglycemic therapy, the proband was discharged after a surgical operation.

On admission, her postprandial glucose was 16.2mmol/L, her fasting plasma glucose was 8.2mmol/L, glycated hemoglobin (HbA1c) was 7.8%, fasting C-peptide was 1.35ng/ml, TG and hs-CRP levels were 0.77mmol/L and 0.95 mg/L, her urine glucose was 4+, with increased of bacteria, leukocytes and erythrocytes in urine. The diabetes autoantibody spectrum (GAD, IA, IA2, IC, and ZnT8) was negative. Other biochemical indices(routine blood test, blood electrolyte analysis, liver function, kidney function, etc.) were within the normal range, with normal weight for age.

The proband was treated with metformin (500 mg a day) and Insulin Detemir (8IU before sleeping). The girl had no significant hypoglycemia during hospitalization. However, combining the results of hs-CRP with fasting C-peptide and diabetes autoantibody spectrum, we believe that the proband lacks the relevant characteristics of T1DM. Considering her age, BMI (19.40 kg/*m*^2^) and record, absence of micro- or macrovascular complications, and no ketosis, we supposed she was not T2DM.

**Fig. 1:**
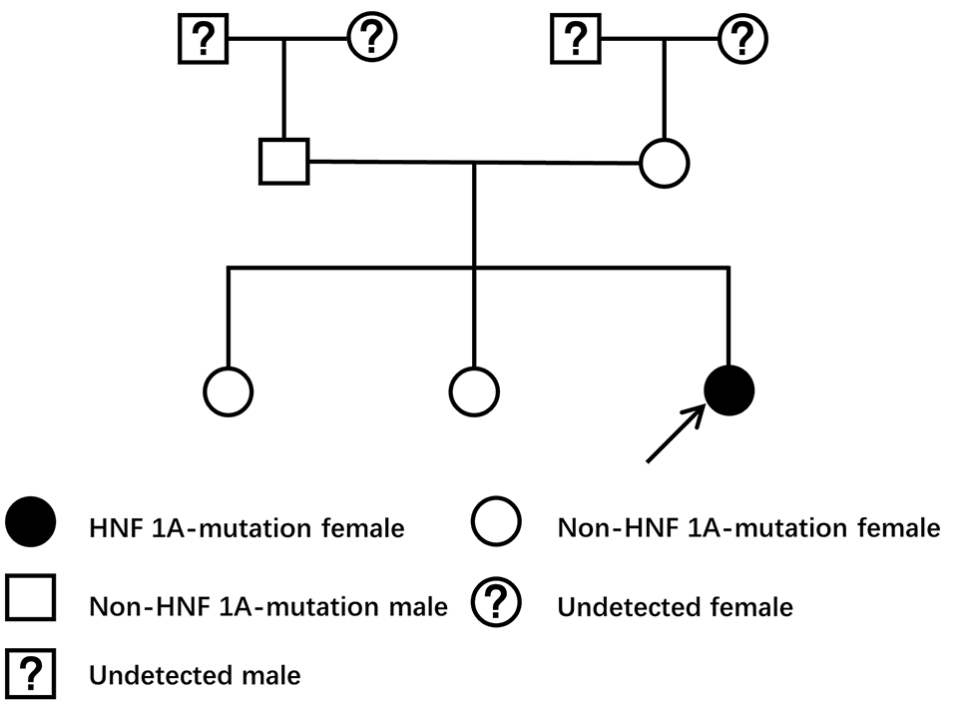
Family genetic pedigree

The proband is the third child in the family. Her maternal mother had no history of gestational diabetes mellitus. However, her grandmother died of diabetic complications. With her parents’ consent, Beijing MyGenostics Institute of Medical Laboratory completed high-throughput sequencing for the proband and her families. MODY3 was diagnosed with heterozygous mutation nomenclature according to accession number NM_0005 45,exon4,of c.788G > A(missense mutations)and protein p.R263H.The gene mutation has clear pathogenicity, which was previously reported in Asia [4, 5]. Furthermore, China has also reported some cases of this gene mutation [6, 7]. According to ACMG guidelines, the variation was preliminarily determined as pathogenic variation(PS2 + PS4 + PM1 + PM2 + PM5 + PP3).Sanger validation shows that her father, mother and two sisters of the proband are non-mutant individuals. Due to monogenic autosomal dominant inheritance, her parents have no relevant gene mutation, it is speculated that her grandmother is not a MODY3 patient.

**Fig. 2:**
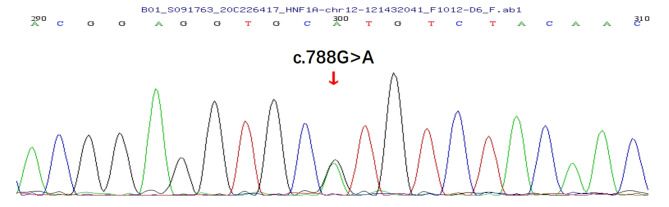
The sequencing chromatogram and position of the proband. The arrow indicates the changed nucleotide base

With ambulatory follow-up by the outpatient clinic of the endocrinology department, she was discharged 6 days later, keeping the treatment plan and maintaining the diabetic diet. After discharge, it was advised to continue with the current treatment. But Insulin Detemir was abandoned by her parents. Treatment was maintained with Metformin 500 mg twice a day which keeps her at a good blood glucose level with 6.7mmol/L fasting and 8.9mmol/L postprandial. We captured her high-throughput sequencing report nearly a month later, which prompts MODY3.

Thus, after the remaining drugs ran out, the patient changed the protocol to glimepiride 4 mg per day. At present, the patient’s fasting blood glucose fluctuates at 5-6mmol / L. Due to poor dietary control, the patient’s postprandial blood glucose is controlled at 9-10mmol / L. The good news is that no hypoglycemic events were found. Her father was very satisfied with the ability to control blood glucose, convenience, and the low price of glimepiride.

## Discussion and conclusions

MODY3 accounts for about 50% of all MODY subtypes [1, 8, 9], generally including significant prandial and fasting hyperglycemia, polyuria, polydipsia, polyphagia, and weight loss. A low renal threshold for glucose reabsorption presents apparent glycosuria. HNF1A-MODY is diabetes not dependent on insulin but sensitivity to sulfonylureas. However, the long course of the disease reveals the pros and cons of the best first-line medications for MODY3 patients.

China was reported to rank first in the amount of diabetes [6]. However, this does not match the few reports on MODY3 in Chinese. This phenomenon implies that the number of MODY3 sufferers is mistakenly classified as T1DM, T2DM or undiagnosed, contributing to indistinguishable symptoms, clinical experience, and affordable testing methods. The exact diagnosis depends on a molecular genetic test, which is costly and not available in community hospitals. Misdiagnosis and missed diagnosis lead to misguided pharmacological intervention resulting in micro- and macro-vascular complications-including 47% of retinopathy,19% nephropathy and 4% neuropathy [9].

During childhood HNF1A mutation carriers could be normoglycemic in plasma but glycosuria. And in several individuals diabetes manifests itself after a phase of neonatal transient hyperinsulinemia hypoglycemia [10, 11]. Once the mother was diagnosed with HNF1A-MODY before pregnancy, the onset of their children was younger than in others. Moreover, the younger one is diagnosed, the shorter their life expectancy will be [12]. The offspring of MODY3 parents have a 50% chance of getting sick due to monogenic autosomal dominant inheritance. Fasting blood glucose increased with age [11]. It is explained by a progressive loss in beta cell function that adds to the physiological impairment in insulin release and glucose tolerance, leading to microvascular complications. But the experiment of John Wiley et al. contradicts this age-related trend [13].

Approximately 80% of MODY patients were divided into T1DM or T2DM [14].However,HNF1A defects increase bile acid synthesis and total cholesterol, HDL-C and LDL-C levels [15].Apolipoprotein M has been suggested as a discriminator between MODY3 and T1DM [16]. HDL-cholesterol differentiates MODY3 from T2DM for a higher level of HNF1A-MODY close to non-diabetic patients [17] but by no means low cardiovascular risk. Furthermore, the evidence shows that the levels of high-sensitivity CRP (hs-CRP) were significantly lower in MODY3 patients compared to T2DM and T1DM or non-diabetes and discriminate MODY3 well from T2DM [18, 19] for mutations in HNF1A. The C-peptide is lower than in non-diabetic patients, but higher than T1DM [15].Combination of hs-CRP with fasting C-peptide, patients with MODY3 distinguished significantly from T1DM(AUC of 1.0) [15]. However, some researchers claim that hs-CRP is not useful to distinguish HNF1A-MODY from young-onset T2DM.It increases during infection and decreases after statin therapy [11]. The positive rate of diabetes autoantibodies affirmed that it distinguishes MODY and T1DM to some extent, deserved high clinical suspicion [20].

HNF1A is a protein with 631 amino acids composed of three functional domains [7]: a dimerization domain (amino acids 1–32),a DNA binding domain (91–276),and a carboxyl-terminal transactivation domain [21].This protein regulates a complex regulatory network important for the differentiation and function of beta cells as a transcription factor. The HNF1A gene is located on chromosome 12q24.31,spanning a region of 120 kb with 10 exons expressed in the pancreas, liver, and kidneys and encodes to regulate the expression of the genes involved in glucose metabolism, glucose transport, and the insulin gene as well as liver-specific genes [22]. It is oriented on the plus strand. The proportions of different types of mutation in functional domains differ. The HNF1A(A) isoform comprises the 10 exons, whereas the HNF1A(B) and HNF1A(C) isoforms contain the first seven and the first six exons, respectively. In terms of diagnostic age, the different types of mutations in the same functional domain and the same type of mutations in different functional domains may be quite different.

The diet without excess saccharides is probably curative when glycosylated hemoglobin is less than 6.5% [11](about one-third of patients were advised non-drug treatment). But low doses of Sulfonylureas (SUs) are the best first-line pharmacological treatment when uncontrollable. The benefits of SUs bind to a membrane protein closely related to the ATP-dependent potassium (K+) channel (KATP channel) in the B cell. Therefore, it closes, causing membrane depolarization, leading to the opening of the voltage-gated calcium (Ca2+) channel and increasing intracellular Ca2 + concentration, increasing insulin secretion. Due to the particular mechanism, hypoglycemia is often associated with sulfonylureas even at low doses [23].Hypersensitivity to sulfonylureas and increasing body weight embarrass adolescent patients who need physical exercise. Sulfonylureas combine the DPP-4 inhibitor linagliptin [24] to reduce glycemic variability and HbA1c without increasing the risk of hypoglycemia or other adverse events [24]. Otherwise, Sulfonylureas are usually effective for several decades, once a severe decrease in B cell insulin production occurs, exogenous insulin is needed.

The receptor of Glucagon-like peptide-1 (GLP-1) makes hypoglycemia rare and allows weight reduction. Furthermore,GLP-1 promotes the generation of pancreatic β-cells and suppresses their apoptosis [25] Although the cAMP pathway is affected by the production of ATP by glucose metabolism, GLP-1 can still amplify the cAMP-mediated insulin secretion pathway. It may be effective for patients with HNF1A diabetes. And liraglutide has been shown to be effective in avoiding hypoglycemia [26]. Many HNF1A patients were treated well with Sulfonylureas (SUs), but there were many reports of successful treatment with GLP-1RA [25, 26]. Thus, GLP-1 often substitutes for SUs when there is frequent hypoglycemia or serious overweight.

A small dose of nateglinide [27] prevents from postprandial hyperglycemia better than glibenclamide and with less stimulation of peak insulin concentrations and less hypoglycemic symptoms in patients with MODY3. Some research reported Dipeptidyl Peptidase-4 inhibitors in patients with HNF1A diabetes when combined with other oral glucose-lowering drugs [28, 29] or with liraglutide as an adjunct therapy to SU and basal insulin [26].

This paper describes a new patient with the HNF mutation in an HNF 1 A (-) family. The physician usually evaluate the possibility of MODY through the family history of HNF mutation. However, this method is not particularly friendly to newly mutated individuals in negative families. In this case, it is suggested that family history should be taken as a supplementary consideration, and more emphasis should be placed on the characteristics of the patients and clinical indicators, to distinguish T1DM from T2DM.

Poor glycaemic control is associated with a two- to three-fold increased risk among MODY3 patients of developing microalbuminuria and retinopathy, respectively. In addition to this, poor blood glucose control can cause a variety of vascular complications. Furthermore, hypoglycemia is dangerous and has long-term damage. It is essential for the control of blood glucose. The genetic test is the only way to diagnose MODY3, leading to optimized treatment. Doctors should be familiar with the key points of distinguishing MODY3 from T1DM and T2DM, especially show interest in patient characteristics, clinical indicators, and genetic history. Suggesting gene testing in time is of great help for patients.

## Data Availability

Not applicable.

## **Abbreviations**

MODY3: A young maturity-onset diabetes of the young type 3
